# Nutritional Composition of the Hehu Field Snail *Cipangopaludina hehuensis*: A Comparison of Open-Air and Greenhouse Paddy Culture Systems

**DOI:** 10.3390/foods15203743

**Published:** 2026-10-21

**Authors:** Jie Chen, Xinyi Qian, Yikai Feng, Deju Zhang

**Affiliations:** 1 Industrial College of Traditional Chinese Medicine and Health, Lishui University, Lishui 323000, China; 2 College of Agriculture and Biotechnology, Lishui University, Lishui 323000, China; lsxyqianxinyi@126.com (X.Q.); lsufyikai@126.com (Y.F.); 3 School of Biological Sciences, The University of Hong Kong, Hong Kong, China; u3005757@connect.hku.hk

**Keywords:** *Cipangopaludina hehuensis*, edible freshwater snail, food composition, fatty acids, vitamins, mineral elements, paddy culture

## Abstract

The Hehu field snail (*Cipangopaludina hehuensis*) is a regional edible gastropod, but its nutritional composition across production systems remains poorly characterized. We evaluated edible muscle from open-air and greenhouse paddies (three independent units per system, each pooling 50 snails) for proximate composition, hydrolyzed amino acids, fatty acids, vitamins, elements, and exploratory volatile profiles. After Welch t tests with Holm correction, greenhouse snails had higher ash (21.77 vs. 19.76 g/100 g dry weight; adjusted *p* = 0.044), whereas moisture, crude protein, crude fat, and quantified amino acids did not differ significantly. Fourteen of 19 quantified fatty acids differed, with their summed concentration higher in open-air snails (1095.76 vs. 817.63 mg/kg wet weight; adjusted *p* = 0.004). The atherogenicity (0.397 vs. 0.489) and thrombogenicity (0.670 vs. 0.719) indices were lower in open-air snails. Vitamin C (254.11 vs. 220.44 mg/kg wet weight) and Fe were higher in open-air snails, whereas vitamin A (0.049 vs. 0.235 mg/kg wet weight), Co, Al, and Pb were higher in greenhouse snails. Acetophenone was the only volatile assignment differing after correction (adjusted *p* = 0.006). These findings establish a nutritional baseline and identify culture-associated differences. Future studies should examine seasonal variation and environmental and dietary contributions.

## 1. Introduction

Edible snails are traditional foods in many regions and can contribute protein, amino acids, fatty acids, and mineral nutrients, although their composition varies among species and production environments [1,2,3,4,5,6,7,8]. The Hehu field snail, *Cipangopaludina hehuensis*, is a freshwater viviparid associated with the mountain rice terraces of Qingyuan County, Zhejiang Province, China. It was described from rice terraces and irrigation ditches in Hehu Town [9] and is locally consumed as a characteristic aquatic food. Production-system-resolved information on the edible-muscle composition of the Hehu snail remains limited.

A comprehensive assessment of edible snail composition can combine proximate composition, amino acids, fatty acids, vitamins, and mineral elements. Proximate composition and hydrolyzed amino acids describe major nutritional components, whereas fatty-acid profiles and vitamins provide additional information on lipid-related and micronutrient characteristics [5,6,7,8,10]. Elemental analysis is also important for evaluating contaminants such as Pb against applicable food-safety limits [11]. Together, these measures allow nutritional and food-safety characteristics to be assessed in the same samples.

Open-air and greenhouse paddy systems expose cultured snails to different combinations of light, temperature, water mixing, natural-food availability, sediment contact, and husbandry. Rice–aquatic animal systems integrate water, sediment, rice plants, periphyton, phytoplankton, detritus, and microbial processes [12,13,14,15,16,17,18]. Light and nutrient availability can alter the fatty-acid composition of freshwater periphyton [19], dietary cyanobacterial exposure can modify muscle metabolites in viviparid snails [20], and periphyton communities can influence metal accumulation in *Cipangopaludina* [21]. Environmental conditions and management practices also affect paddy-field snails and the muscle composition of cultured aquatic animals [22,23,24]. Together, these findings suggest that differences in production environment can be reflected in the edible-muscle composition of Hehu snails.

Volatile compounds are another component of aquatic-food characterization. Headspace solid-phase microextraction–gas chromatography–mass spectrometry (HS-SPME-GC-MS) is widely used to profile volatile compounds in shellfish and other aquatic products [25,26,27,28,29]. We therefore included volatile profiling as a complementary analysis to examine whether the two production systems were associated with differences in the instrumental volatile profile.

**Figure 1:**
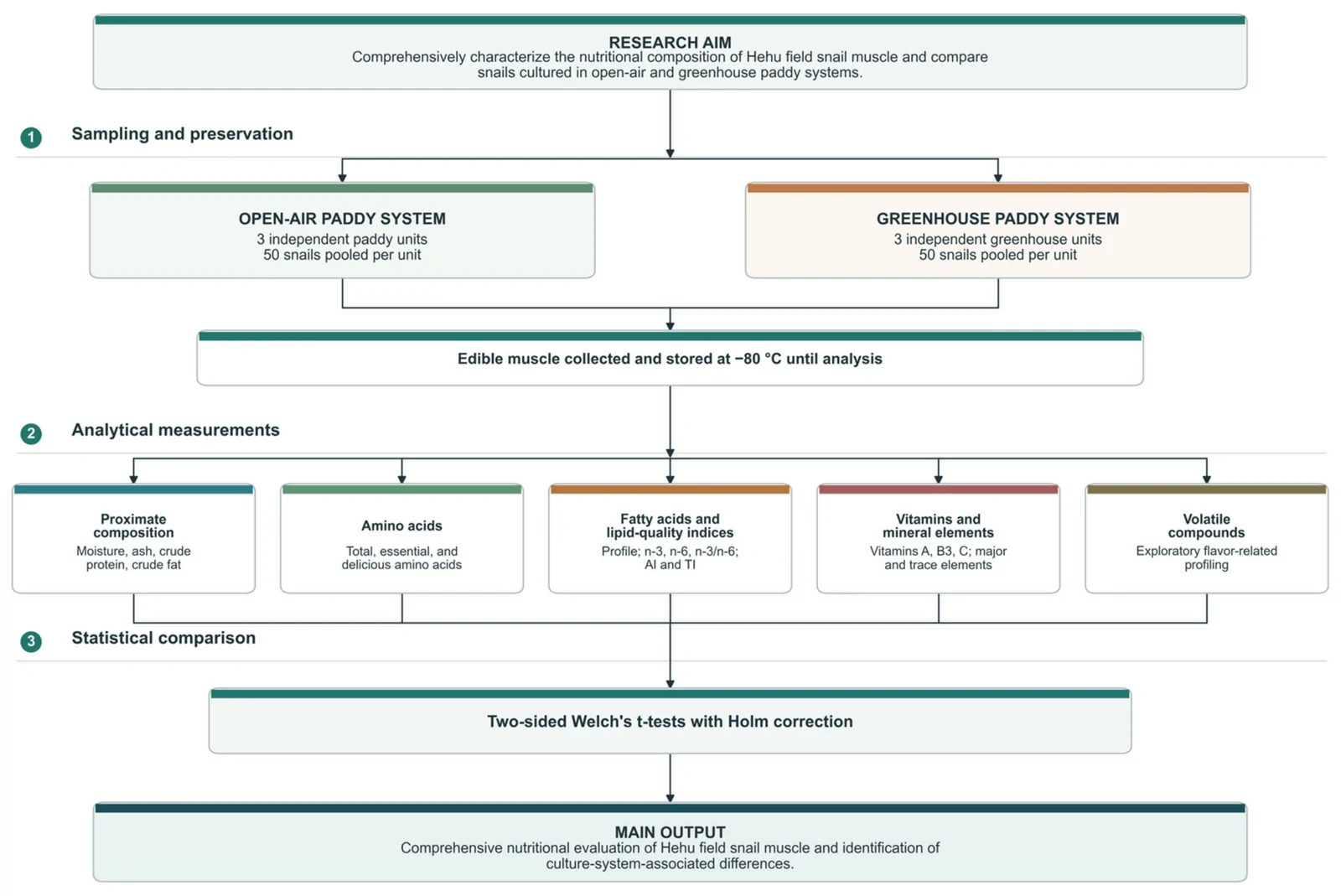
Overview of the study design and analytical workflow.

To establish a comprehensive nutritional profile of *C. hehuensis* and compare the two production systems, we examined edible muscle from open-air and greenhouse paddies. Each system was represented by three independent paddy units of similar nominal area and water depth. Proximate composition, hydrolyzed amino acids, quantified fatty acids, vitamins, and mineral elements were assessed, while exploratory volatile profiling provided complementary information on potential flavor-related characteristics. The sampling design and analytical workflow are summarized in Figure 1.

## 2. Materials and Methods

### 2.1. Experimental Design and Culture Systems

Two practical production systems were compared: open-air paddy culture and greenhouse paddy culture. Each system comprised three independent paddy units, each approximately 666.7 m^2^ with a nominal water depth of 0.30 m. The three greenhouse units were located in three separate greenhouses, while the three open-air units were spatially independent paddies at the same production site. Water exchange in both systems depended mainly on natural inflow and drainage.

The systems had the same nominal unit area and water depth but differed in greenhouse cover, outdoor exposure, and associated husbandry. The paddy unit was treated as the biological and statistical unit.

### 2.2. Culture Conditions and Management Records

Water-quality and husbandry conditions were recorded during culture. Table S1 summarizes the production-period ranges and management records for water temperature, pH, dissolved oxygen, nutrient concentrations (NH_4_^+^-N, NO_2_^−^-N, NO_3_^−^-N, and total phosphorus), transparency, chlorophyll-a, stocking density, feed input and composition, fertilization and liming, and rice cultivation management. Both systems received a mixed feed comprising rice bran (50%), wheat bran (20%), soybean meal or bean cake (20%), and corn meal (10%). Feeding rates and schedules varied with estimated snail biomass and natural-food availability. Natural food included periphyton, phytoplankton, detritus, and benthic microorganisms.

### 2.3. Sample Collection and Preparation

Market-sized Hehu field snails were collected on 10 August 2025, from Zhejiang Shuoyuan Agricultural Technology Co., Ltd., Qingyuan County, Zhejiang Province, China (27°46′34″ N, 119°17′24″ E; altitude, 1108 m). Representative specimens had a body weight of 2.33 ± 0.58 g, shell height of 2.27 ± 0.06 cm, and shell width of 1.77 ± 0.06 cm. Fifty snails were randomly collected from each of the six independent paddy units. After shell removal, edible muscle tissue was carefully dissected to exclude the digestive tract and other visceral tissues. Muscle from the 50 snails within each unit was pooled and homogenized to form one biological replicate; samples from different units were processed and analyzed separately. Each system therefore comprised three biological replicates and 150 snails, giving 300 snails in total. For laboratory analysis, subsamples were stored at −80 °C to minimize biochemical and microbial changes.

The sampled Hehu population is referred to as *C. hehuensis* because this name was used in the sampling records and is consistent with the 2016 species description and earlier local nutritional literature [9,30]. Recent studies have treated *C. hehuensis* as a junior synonym of *Margarya chinensis* [31] or identified the Hehu population as *C. cathayensis* [32]. We retain the sampling name for consistency without proposing a new taxonomic determination.

### 2.4. Proximate Composition

Moisture, ash, crude fat, and crude protein were determined according to Chinese national food-safety standards. Moisture was measured on a fresh-weight basis by direct drying according to GB 5009.3-2016 [33]; ash was measured on a dry-weight basis by direct ashing according to GB 5009.4-2016 [34]; crude protein was measured on a dry-weight basis by the Kjeldahl method according to GB 5009.5-2016 [35]; and crude fat was measured on a dry-weight basis by Soxhlet extraction according to GB 5009.6-2016 [36]. Moisture is expressed as percentage fresh weight, whereas crude protein, crude fat, and ash are expressed as g/100 g dry weight or percentage dry weight, as appropriate.

### 2.5. Amino Acid Analysis

Amino-acid composition was determined using a commercial high-performance liquid chromatography (HPLC) procedure developed with reference to GB 5009.124-2016 [37]. Approximately 0.5000 g of homogenized muscle was mixed with 10 mL of 6 mol/L HCl and hydrolyzed at 110 °C for 8 h. After filtration, 2 mL of 50% ethanol containing 0.1 mol/L HCl was added; the extract was sonicated for 30 min and centrifuged at 12,000 r/min for 5 min. A 1 mL aliquot of supernatant was frozen at −80 °C, lyophilized, reconstituted in 1 mL of amino-acid sample diluent, mixed, and filtered through a 0.22 μm membrane. Amino acids were analyzed on a Vanquish HPLC system (Thermo Fisher Scientific, Waltham, MA, USA) fitted with a Diamonsil AAA column (5 μm, 250 × 4.6 mm), using a 10 μL injection volume, a flow rate of 1.0 mL/min, a column temperature of 40 °C, and UV absorbance detection at 254 nm. Commercial amino-acid standards (Anpel-Trace Standard Technical Services (Shanghai) Co., Ltd., Shanghai, China) were used for identification and quantification, and results are reported as mg/100 g wet weight (WW). Tryptophan was not determined because it is degraded during strong-acid hydrolysis. Because performic-acid oxidation was not used, methionine and cysteine are reported as acid-hydrolysate values and may be underestimated due to incomplete recovery. TAA, EAA, DAA, EAA/TAA, and DAA/TAA were calculated from the quantified analytes. EAA comprised Leu, Ile, Val, Thr, Lys, Met, Phe, and His, whereas DAA comprised Asp, Glu, Gly, and Ala. Asn and Gln were not quantified separately under the hydrolysis conditions used.

### 2.6. Fatty Acid Analysis

Fatty-acid composition was determined according to GB 5009.168-2016 using hydrolysis, extraction, and fatty-acid methyl ester analysis [38]. Approximately 2.0 g of homogenized muscle was mixed with 100 mg of pyrogallol, 2 mL of 95% ethanol, and 4 mL of water. After adding 5 mL of aqueous ammonia, the mixture was hydrolyzed at 70–80 °C for 20 min with intermittent shaking. Following cooling, 10 mL of 95% ethanol was added, and lipids were extracted four times with diethyl ether–petroleum ether (1:1, *v*/*v*). The combined extracts were evaporated to dryness. The extracted lipids were saponified with 8 mL of 2% NaOH in methanol at 80 ± 1 °C until the oil droplets disappeared, followed by methylation with 7 mL of 15% boron trifluoride in methanol for 2 min. The fatty-acid methyl esters were extracted with 20 mL of n-heptane and dried over anhydrous sodium sulfate. Samples were not dried before hydrolysis or extraction. The methyl esters were analyzed using a gas chromatography system (Trace 1300, Thermo Fisher Scientific, Waltham, MA, USA) equipped with a TR-FAME capillary column (100 m × 0.25 mm × 0.20 μm). Helium was the carrier gas. Fatty acids were identified and quantified with a 37-component fatty-acid methyl ester standard mixture. Quantification was performed using six-level external-standard calibration without an internal standard. Concentrations were calculated from the calibrated fatty-acid methyl ester concentration (mg/L), the final extract volume (mL), sample mass (g), and the corresponding methyl ester-to-fatty acid molecular-weight conversion factor. Individual fatty acid concentrations are expressed as mg/kg wet weight (WW), equivalent to μg/g wet weight. ΣQFA, ΣQSFA, ΣQMUFA, ΣQPUFA, ΣQUFA, and QUFA/QSFA were calculated from the quantified target fatty acids. Nineteen analytes from the 37-component target panel were quantified, and sums and ratios were calculated separately for each biological replicate. Fatty-acid nutritional measures were calculated for each replicate: Σn-3 = C18:3(n-3) + EPA; Σn-6 = C18:2(n-6) + C20:2(n-6) + C20:4(n-6); and n-3/n-6. The atherogenicity index (AI) was (C12:0 + 4 × C14:0 + C16:0)/(ΣQMUFA + Σn-6 + Σn-3), and the thrombogenicity index (TI) was (C14:0 + C16:0 + C18:0)/(0.5 × ΣQMUFA + 0.5 × Σn-6 + 3 × Σn-3 + Σn-3/Σn-6) [39]. C12:0 was not detected and was excluded from the calculation. All reported fatty-acid sums therefore represent only these 19 quantified targets, not total fatty-acid content.

### 2.7. Vitamin Analysis

Vitamin C, vitamin B3, and vitamin A were determined by high-performance liquid chromatography with external-standard calibration. Vitamin A was determined according to GB 5009.82-2016 [40], and vitamin C was determined with reference to GB 5009.86-2016 [41]. Vitamin B3 was determined using the laboratory HPLC procedure described below. For vitamin C, homogenized samples were extracted with 5% metaphosphoric acid, centrifuged, filtered through a 0.45 μm membrane, and analyzed using a Vanquish UHPLC system (Thermo Fisher Scientific, Waltham, MA, USA) with a Nova Atom C18 column (250 mm × 4.6 mm, 5 μm). The mobile phase was 0.1% oxalic acid, and detection was at 266 nm. For vitamin B3, samples were extracted with 0.1 mol/L hydrochloric acid, treated with zinc sulfate, centrifuged, filtered through a 0.45 μm membrane, and analyzed with the same UHPLC system and column at 270 nm. For vitamin A, samples were enzymatically hydrolyzed with lipase, extracted with acetone and ether, dried under nitrogen, reconstituted in methanol, filtered through a 0.22 μm membrane, and analyzed by HPLC. Vitamins are reported as mg/kg wet weight (WW).

### 2.8. Mineral Element Analysis

Mineral elements were determined according to GB 5009.268-2016 after nitric-acid digestion followed by inductively coupled plasma optical emission spectrometry (ICP-OES) or inductively coupled plasma mass spectrometry (ICP-MS) analysis [42]. Homogenized muscle samples were digested with 3 mL of nitric acid in polytetrafluoroethylene vessels using a programmed microwave digestion (10 min to 150 °C, 2 min at 150 °C; 18 min to 240 °C, 30 min at 240 °C), transferred to 25 mL volumetric flasks, and made to volume with 1% nitric acid. Ca, K, Na, Mg, S, Fe, Zn, Co, Mo, Cu, Mn, Se, Ge, Pb, B, and Al were measured using ICP-OES (iCAP 7200 HS Duo, Thermo Fisher Scientific, Waltham, MA, USA) and ICP-MS (iCAP RQ, Thermo Fisher Scientific, Waltham, MA, USA). A reagent-blank series was processed in parallel. The analytical laboratory also measured a quality-control material designated GSB-12 in triplicate; for all 16 reported elements, the measured values were within the reference value ± uncertainty ranges supplied in the analytical worksheet. Elemental concentrations are reported on a dry-weight basis throughout. For the food-safety discussion, greenhouse Pb and Al concentrations were additionally converted to fresh-weight equivalents using that group’s mean moisture content (80.69%; dry-matter fraction = 0.1931). The Pb estimate was compared with the applicable fresh-weight limit, while the elevated Al estimate was considered in the context of potential dietary exposure.

### 2.9. Exploratory Volatile Profiling

Motivated by local reports of the savory taste of Hehu snail broth, we analyzed volatile compounds in uncooked edible muscle as a complement to the nutritional measurements, rather than as a direct measure of broth umami. Volatile compounds were analyzed by headspace solid-phase microextraction coupled with gas chromatography–mass spectrometry (HS-SPME-GC-MS), with reference to previous studies of aquatic products and shellfish [25,28,29]. Homogenized muscle sample (2.0 g) was mixed with saturated sodium chloride solution [salt:water = 1:3 (g:mL)] and transferred to a 20 mL headspace vial. Volatiles were extracted with a 65 μm DVB-PDMS SPME fiber (Supelco, Bellefonte, PA, USA) at 60 °C for 30 min and thermally desorbed in the GC injection port (Agilent Technologies, Santa Clara, CA, USA). GC-MS analysis used a DB-5MS capillary column (30 m × 0.25 mm × 0.25 μm) with helium as the carrier gas at 1.0 mL/min. The oven program was 35 °C for 1 min, then 140 °C at 5 °C/min for 1 min, followed by 230 °C at 8 °C/min for 5 min. The ion-source temperature was 200 °C, and mass spectra were acquired over m/z 35–350. Compounds were tentatively assigned by matching mass spectra against the NIST 2023 library, and raw integrated peak areas (counts·min) were exported for each biological replicate. Repeated peaks sharing a valid CAS number were summed within sample. Candidate assignments were retained when they occurred in all six samples, had a valid CAS number, and had a reverse-library-match score ≥ 800 in every sample; common siloxane, phthalate, and phosphate-ester backgrounds were excluded. Of 46 assignments common to all six samples, 21 met these criteria. Screening details, match scores, raw areas, and inclusion decisions are provided in Table S2. Group comparisons used raw peak areas and were therefore semi-quantitative, as no internal standard or calibration curve was used. The original GC-MS total ion chromatograms for all six paddy samples are provided in Figure S1.

### 2.10. Statistical Analysis

**Figure 2:**
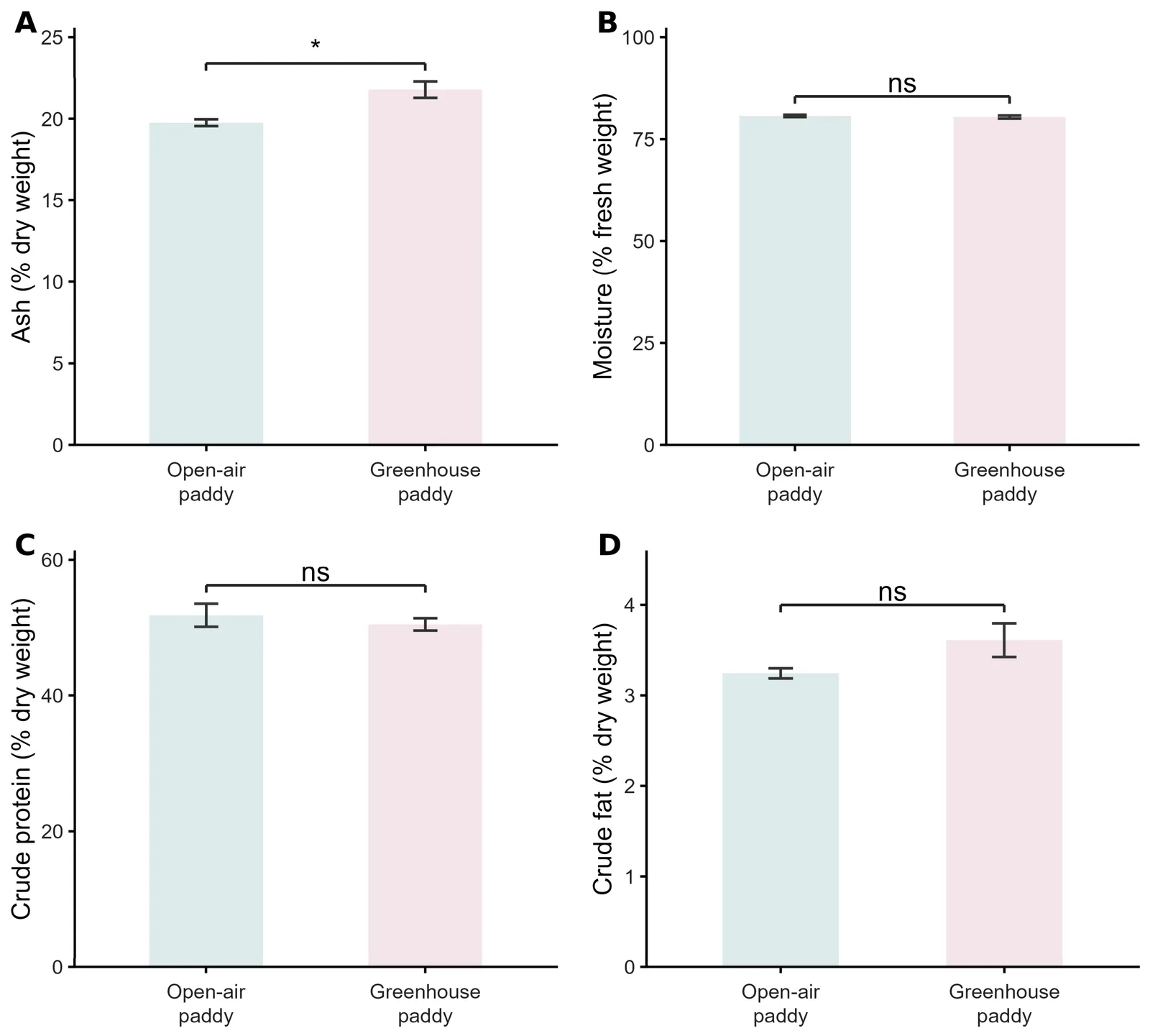
Proximate composition of Hehu field snail muscle across culture systems. (**A**) Ash, (**B**) moisture, (**C**) crude protein, and (**D**) crude fat. Moisture is expressed on a fresh-weight basis; the other components are expressed on a dry-weight basis. Bars and error bars show means ± SD (n = 3 independent biological replicates per system). *p* values are from two-sided Welch t tests with Holm correction across the four components. Adjusted *p* values were 0.044 (**A**), 0.621 (**B**), 0.621 (**C**), and 0.191 (**D**). * indicates adjusted *p* < 0.05; ns, not significant.

**Figure 3:**
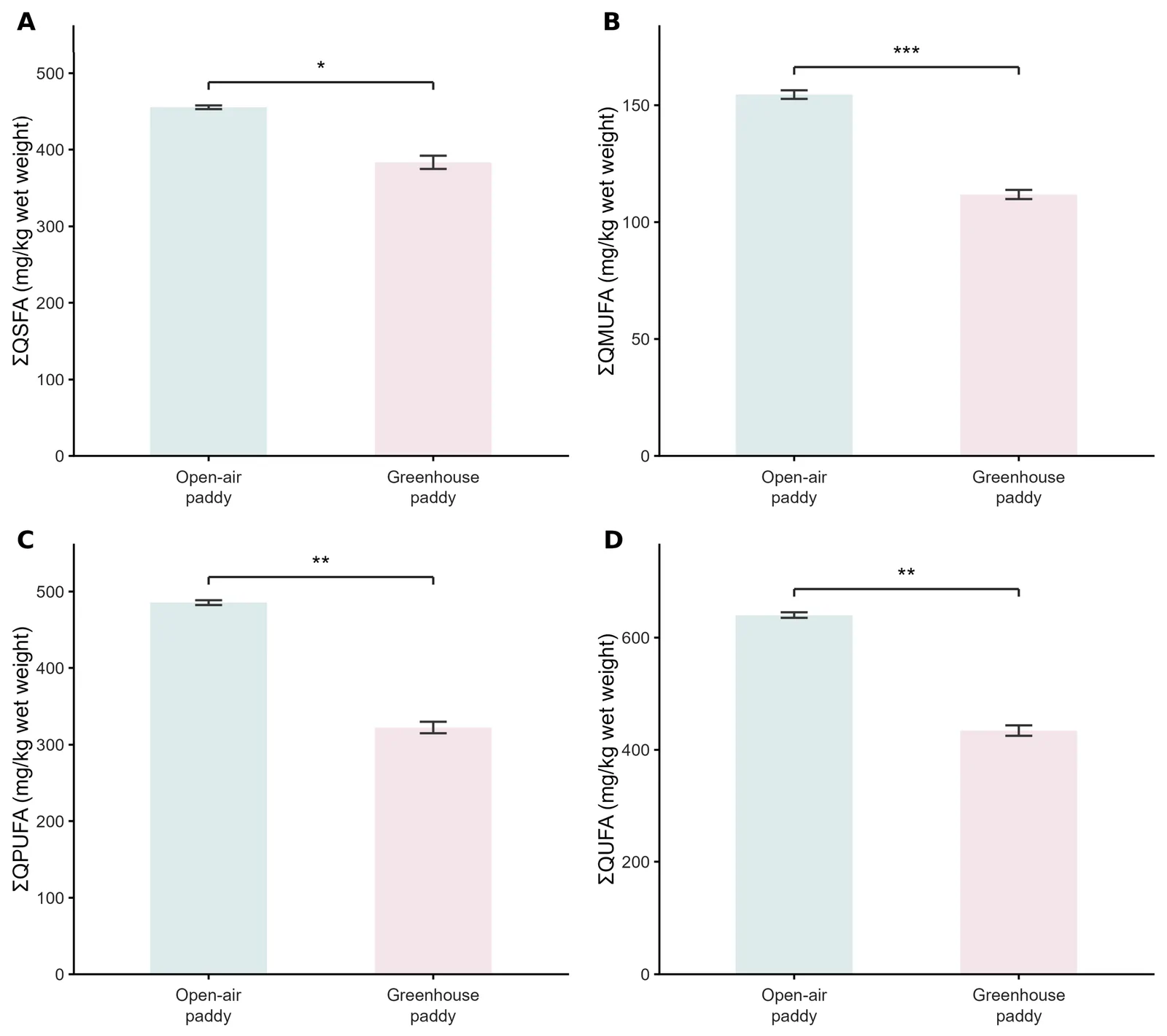
Comparison of summary indices calculated from quantified target fatty acids in the muscle of Hehu field snail across culture systems. (**A**) Sum of quantified saturated fatty acids (ΣQSFA); (**B**) monounsaturated fatty acids (ΣQMUFA); (**C**) polyunsaturated fatty acids (ΣQPUFA); and (**D**) unsaturated fatty acids (ΣQUFA). Bars and error bars show means ± SD (n = 3 independent biological replicates per system). *p* values are from two-sided Welch t tests with Holm correction across the 25 comparisons in Table 2. All indices are expressed as mg/kg wet weight and represent sums of quantified target analytes, not total fatty-acid content or crude fat. Adjusted *p* values were 0.020 (**A**), <0.001 (**B**), 0.002 (**C**), and 0.001 (**D**). * adjusted *p* < 0.05; ** adjusted *p* < 0.01; *** adjusted *p* < 0.001.

**Figure 4:**
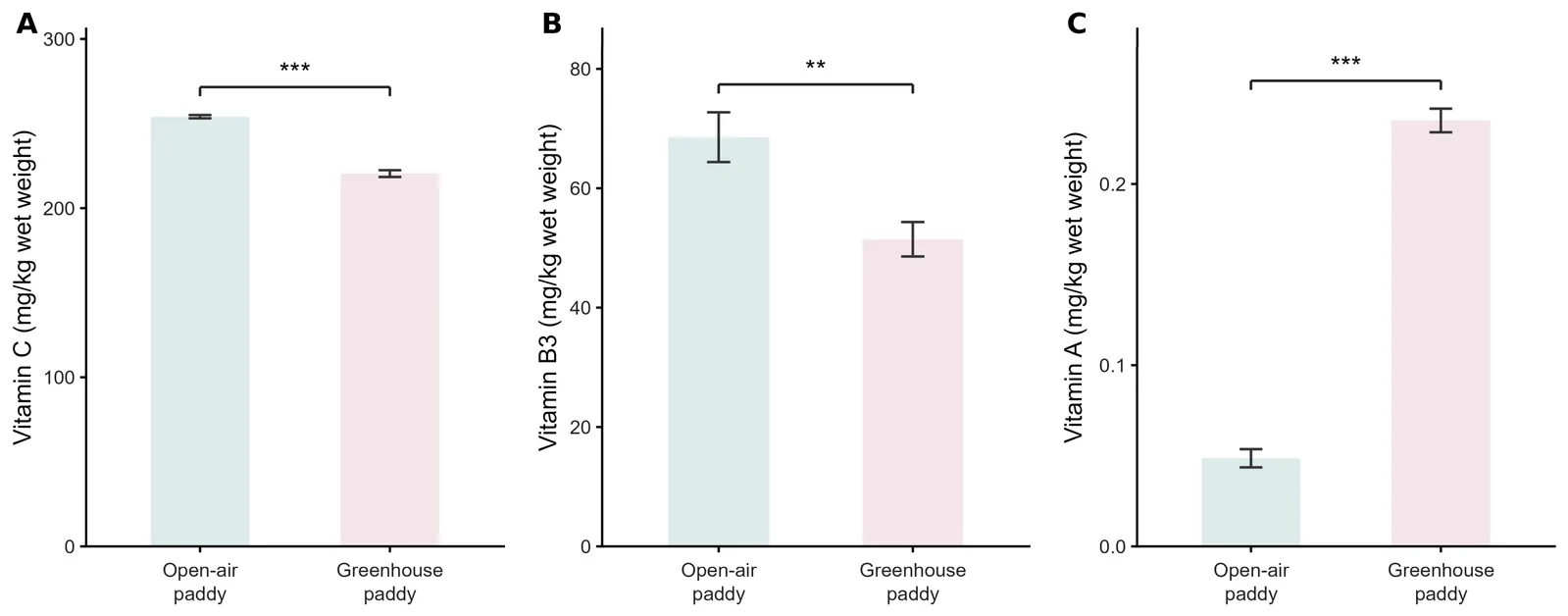
Vitamin contents in Hehu field snail muscle across culture systems. (**A**) Vitamin C, (**B**) vitamin B3, and (**C**) vitamin A. Contents are expressed as mg/kg wet weight. Bars and error bars show means ± SD (n = 3 independent biological replicates per system). *p* values are from two-sided Welch t tests with Holm correction across the three vitamins. Adjusted *p* values were <0.001 (**A**), 0.006 (**B**), and <0.001 (**C**). ** adjusted *p* < 0.01; *** adjusted *p* < 0.001.

Data are presented as means ± SD from three independent biological replicates per system, with each paddy unit treated as the statistical unit. Culture system (open-air versus greenhouse paddy) was the only fixed effect assessed; no random effects were fitted because each unit contributed one pooled biological replicate. Between-system differences were evaluated with two-sided Welch t tests, followed by Holm correction within each analytical category. For fatty-acid analyses, Holm correction was applied separately to 25 fatty-acid composition comparisons and five exploratory nutritional measures. Adjusted *p* < 0.05 was considered statistically significant; adjusted *p* ≥ 0.05 indicates that a difference was not detected and does not demonstrate equivalence. Adjusted *p* values are reported in Table 1, Table 2, Table 3 and Table 4 and Figure 2, Figure 3 and Figure 4. Analyses were performed in R (version 4.6.1; R Foundation for Statistical Computing, Vienna, Austria).

## 3. Results

### 3.1. Proximate Composition

Ash was higher in greenhouse paddy snails than in open-air paddy snails (21.77 ± 0.50 versus 19.76 ± 0.21 g/100 g dry weight; Holm-adjusted *p* = 0.044; Figure 2). Moisture averaged 80.40 ± 0.38% and 80.69 ± 0.28%, respectively. Crude protein was 50.47 ± 0.91 versus 51.81 ± 1.69 g/100 g dry weight, and crude fat was 3.61 ± 0.18 versus 3.24 ± 0.06 g/100 g dry weight. Moisture, crude protein, and crude fat did not differ significantly after correction for the four comparisons.

### 3.2. Amino Acid Composition

Seventeen amino acids were quantified in muscle hydrolysates (Table 1). Glu, Gly, Ser, and Asp were among the most abundant measured amino acids in both systems. TAA averaged 1440.50 ± 43.37 mg/100 g wet weight in open-air paddy snails and 1479.79 ± 68.75 mg/100 g wet weight in greenhouse paddy snails. Asn and Gln were not quantified separately under the hydrolysis conditions, and Trp was not measured.

None of the 17 individual amino acids or five related indices differed significantly after Holm correction across 22 comparisons. EAA/TAA was 26.65 ± 0.32% in the open-air group and 26.24 ± 1.06% in the greenhouse group; DAA/TAA was 49.56 ± 0.79% and 50.09 ± 1.71%, respectively.

### 3.3. Fatty Acid Composition

Nineteen target fatty acids were quantified (Table 2). The sum of quantified fatty acids (ΣQFA) was higher in open-air paddy snails than in greenhouse paddy snails (1095.76 ± 7.02 versus 817.63 ± 17.45 mg/kg wet weight). Fourteen individual fatty acids differed significantly after Holm correction across the 19 analytes and six derived indices. Thirteen were higher in the open-air group, whereas trans-C18:1 showed the opposite pattern, with a higher concentration in the greenhouse group (adjusted *p* = 0.020). EPA did not differ significantly (25.02 ± 0.40 versus 22.31 ± 1.82 mg/kg wet weight; adjusted *p* = 0.235).

All six fatty-acid summary measures were higher in the open-air group (Table 2), and four are illustrated in Figure 3. The quantified saturated (ΣQSFA), monounsaturated (ΣQMUFA), and polyunsaturated fatty-acid sums (ΣQPUFA) were 455.66 ± 2.41, 154.53 ± 1.83, and 485.57 ± 3.12 mg/kg wet weight, respectively, compared with 383.41 ± 8.63, 111.78 ± 1.95, and 322.43 ± 7.50 mg/kg wet weight in the greenhouse group. The quantified unsaturated-to-saturated ratio (QUFA/QSFA) was 1.405 ± 0.007 versus 1.133 ± 0.011. These sums are restricted to the 19 quantified targets and do not represent total fatty-acid content.

For the fatty-acid nutritional measures, Σn-3 and Σn-6 concentrations were higher in open-air snails, but the n-3/n-6 ratio was lower (0.111 ± 0.002 vs. 0.140 ± 0.008). Both AI (0.397 ± 0.004 vs. 0.489 ± 0.004) and TI (0.670 ± 0.006 vs. 0.719 ± 0.009) were also lower in the open-air group. All five measures differed after Holm correction within this exploratory set (adjusted *p* ≤ 0.038; Table 2).

### 3.4. Vitamin Composition

All three vitamins differed significantly between systems after Holm correction (Figure 4). Vitamin C and vitamin B3 were higher in open-air paddy snails (254.11 ± 0.98 and 68.56 ± 4.16 mg/kg wet weight) than in greenhouse paddy snails (220.44 ± 1.99 and 51.43 ± 2.92 mg/kg wet weight). Vitamin A showed the opposite pattern, with 0.049 ± 0.005 mg/kg wet weight in the open-air group and 0.235 ± 0.007 mg/kg wet weight in the greenhouse group.

### 3.5. Elemental Composition

Four of the 16 measured elements differed significantly after Holm correction (Table 3). Fe was higher in open-air paddy snails than in greenhouse paddy snails (2.87 ± 0.08 versus 2.20 ± 0.12 g/kg dry weight). Co, Al, and Pb were higher in the greenhouse group: 0.908 ± 0.026 versus 0.623 ± 0.002 mg/kg, 7240 ± 140 versus 5560 ± 130 mg/kg, and 2.32 ± 0.09 versus 1.57 ± 0.06 mg/kg dry weight, respectively. No significant differences were detected for the remaining elements.

### 3.6. Exploratory Volatile Profile

Across the six paddy samples, 46 compound-name/CAS assignments were common to all samples after repeated peaks were collapsed within sample. An objective screening procedure based on prevalence, valid CAS identity, reverse library-match score (≥800 in every sample), and exclusion of common analytical contaminants retained 21 tentative assignments for exploratory comparison (Table S2 and Table 4). After Holm correction across these 21 tests, acetophenone was the only assignment that differed significantly, with a higher raw integrated peak area in open-air than greenhouse snails (0.667 ± 0.023 vs. 0.403 ± 0.028 × 10^6^ counts·min; adjusted *p* = 0.006). Benzaldehyde showed a similar directional difference but did not remain significant after correction (adjusted *p* = 0.052). Overall, the screened volatile profile was comparatively stable between systems.

## 4. Discussion

Differences between the two culture systems were most evident in the quantified fatty-acid profile, vitamins, and selected elements. Moisture, crude protein, crude fat, and hydrolyzed amino acids showed no significant between-system differences, while only one tentative volatile assignment remained significant after multiplicity correction. Thus, culture-system differences were confined to specific components.

Greenhouse snails had higher ash content, but this was not accompanied by uniformly higher concentrations of individual elements: Fe was higher in open-air snails, whereas Co, Al, and Pb were higher in greenhouse snails. Ash represents total inorganic residue, whereas individual elemental concentrations can vary with environmental exposure, diet, and tissue accumulation. Variation in mineral composition has also been reported among edible mollusks and other aquatic foods [3,5,8].

Although the systems had similar nominal paddy area and water depth, greenhouse cover and husbandry were associated with different thermal and food conditions. Recorded water temperatures were 21.5–29.0 °C in greenhouse paddies and 18.5–26.5 °C in open-air paddies (Table S1). Both systems received the same formulated feed, but supplemental input was approximately 1–2% of estimated snail biomass in open-air paddies and 1.5–2.5% in greenhouse paddies. Open-air units also had higher recorded chlorophyll-a ranges (20–45 versus 15–35 µg/L) and qualitatively greater periphyton and phytoplankton abundance (Table S1). These differences could affect natural-food composition and exposure to sediment-associated elements [16,17,19,20,21]. Because actual feed intake and natural-food composition were not measured, the respective contributions of diet and environment remain uncertain.

The hydrolyzed amino-acid composition was comparatively consistent across the two culture systems: none of the 17 measured amino acids or derived indices remained significant after Holm correction. Glu, Gly, Ser, and Asp were among the most abundant amino acids in both groups. Glu and Asp, which are associated with umami, together accounted for approximately 28.0% and 26.6% of measured TAA in open-air and greenhouse snails, respectively (calculated from group means). The broader DAA fraction (Asp, Glu, Gly, and Ala) represented 49.56 ± 0.79% and 50.09 ± 1.71% of TAA, respectively, without a significant between-system difference (Table 1). These results describe acid-hydrolysate composition, not the free amino acids that contribute directly to perceived taste [37].

The essential-amino-acid fraction was similarly consistent, accounting for 26.65 ± 0.32% and 26.24 ± 1.06% of total quantified amino acids in open-air and greenhouse snails, respectively (Table 1). Threonine and leucine were the most abundant quantified essential amino acids, whereas methionine was measured at a lower concentration. Ghosh et al. [1] reported a higher essential-amino-acid proportion (39.7%) in farmed *Pomacea canaliculata* and noted relatively low methionine; however, differences in species, amino-acid recovery, and inclusion of tryptophan prevent direct comparison of protein quality. A chemical amino-acid score requires a reliable profile of all essential amino acids expressed per gram of protein and assessed against an appropriate FAO reference pattern [10]. Because tryptophan was not measured and methionine and cysteine may have been incompletely recovered, neither an overall chemical score nor the first-limiting amino acid could be determined reliably.

The targeted fatty-acid profile showed a stronger association with culture system. Fourteen of the 19 quantified fatty acids differed after multiplicity correction; 13 were higher in open-air snails, whereas trans-C18:1 was higher in greenhouse snails. Open-air snails also had higher quantified saturated, monounsaturated, polyunsaturated, and unsaturated fatty-acid sums and a higher QUFA/QSFA ratio. These differences do not conflict with the lack of a significant crude-fat difference: Soxhlet extraction measures bulk extractable lipids on a dry-weight basis, whereas GC quantified a limited panel of fatty acids on a wet-weight basis. The recorded greater availability of periphyton and phytoplankton in open-air paddies, together with the higher supplemental feed input in greenhouse paddies (Table S1), suggests different relative contributions from natural and formulated lipid sources. Periphyton fatty-acid composition responds to light and nutrient conditions [19], and an experiment with *Bellamya aeruginosa* found that warming reduced periphyton PUFA from 13.32% to 9.90%, alongside a decrease in snail PUFA from 40.14% to 36.27% [43]. Although consistent with a possible dietary contribution, that study used different analytical measures, and neither actual intake nor source-specific fatty acids were measured here; the mechanism therefore remains unconfirmed. The fatty-acid nutritional measures showed higher quantified n-3 and n-6 and lower AI and TI in open-air snails, but a lower n-3/n-6 ratio than in greenhouse snails. As these indices derive from only 19 quantified targets, they should not be interpreted as a complete fatty-acid profile or evidence of overall dietary health benefits.

Two odd-chain saturated fatty acids, C15:0 and C17:0, were also detected in both systems. Their concentrations were 21.29 and 26.41 mg/kg wet weight in open-air snails and 15.14 and 19.52 mg/kg in greenhouse snails (Table 2), together accounting for approximately 4.35% and 4.24% of the quantified fatty-acid panel, respectively. These were minor components, and odd-chain fatty acids have also been reported in *Bellamya aeruginosa*, with C15:0 and C17:0 representing approximately 3.1–4.3% and 3.0–3.2% of profiled fatty acids, respectively [43]. The percentages are not directly comparable because the analytical panels differed. Microorganisms can synthesize odd-chain fatty acids from propionyl-CoA [44], making microbial biofilms and natural food plausible sources, but dietary uptake and endogenous metabolism were not examined separately. Their origin and the reasons for between-system differences therefore remain uncertain.

The vitamin results also varied by production system: vitamin C and vitamin B3 were higher in open-air snails, whereas vitamin A was higher in greenhouse snails. Dietary and metabolic differences may contribute, although these were not measured directly. Elemental concentrations require additional consideration because snails may encounter metals through sediment contact, periphyton or detritus ingestion, irrigation water, and supplemental feed [21]. These pathways could contribute to the higher Al and Pb levels in greenhouse snails, but the respective sources were not determined; the concentrations nevertheless warrant attention in production monitoring.

For greenhouse snails, the mean Pb concentration of 2.32 mg/kg dry weight was equivalent to approximately 0.448 mg/kg fresh weight, based on the measured mean moisture content of 80.69%. This was below the 1.0 mg/kg Pb limit specified by GB 2762-2025 for fresh and frozen aquatic animals other than fish, crustaceans, and bivalves, with viscera removed [11]. The estimated Al concentration was approximately 1398 mg/kg fresh weight. GB 2762-2025 does not specify an Al limit for this food category, and the absence of such a limit does not establish safety. For context, a hypothetical 100 g serving at this estimated concentration would contain about 140 mg Al, exceeding the 120 mg/week corresponding to the JECFA provisional tolerable weekly intake (2 mg/kg body weight/week) for a 60 kg adult [45]. This comparison is illustrative rather than a formal risk assessment: actual consumption, processing effects, Al bioavailability, and independent confirmation of the elevated concentration were not evaluated.

The Fe values also require context because they exceeded those in a previous study of snails from Hehu. That study reported 5.263 ± 0.172 mg Fe/100 g fresh edible flesh after two days of clean-water holding, equivalent to approximately 0.217 g/kg dry weight using its reported moisture content [30]; the present means were 2.20–2.87 g/kg dry weight. Differences in sample preparation and depuration, analytical methods, diet, or environmental exposure may help explain the discrepancy. In flooded paddy soils, reductive dissolution of Fe (oxyhydr)oxides can release Fe into porewater [46], potentially increasing exposure through water, sediment-associated particles, or natural food. Because an equivalent clean-water holding step was not documented and environmental Fe or its contribution to muscle concentrations was not measured, the reason for the higher Fe values remains uncertain.

Compared with the nutrient and element measurements, volatile profiles differed little between culture systems. Of the 21 retained tentative assignments, only acetophenone remained significant after Holm correction; benzaldehyde approached, but did not reach, the adjusted threshold. The volatile results therefore provide complementary information on potential flavor-related compounds but do not establish sensory differences between culture systems. Because no internal standard or calibration curve was used, the peak-area data were exploratory and semi-quantitative; mass-spectral assignments also require confirmation with retention indices or authentic standards [25,26,27,28,29]. Although the higher acetophenone signal in open-air snails could contribute to aroma, its sensory relevance, particularly for cooked snail broth, cannot be determined without quantitative measurements and sensory evaluation.

Interpretation is limited by sampling at one high-altitude site and a single harvest time, with three independent paddy units per system. The 50 snails pooled within each unit did not provide additional independent replicates, and the small number of units reduced power to detect modest differences; non-significant results therefore should not be taken as evidence of equivalence. Without paired measurements of dietary intake or environmental exposures, the observed associations cannot be assigned to individual causal factors.

Further studies could test whether these nutritional patterns persist across sites and seasons using more independently replicated open-air and greenhouse paddies. Concurrent measurements of formulated feed, natural food, water, and sediment would help identify potential contributors to the fatty-acid and elemental differences, especially those involving Al, Pb, and Fe. Analysis of free amino acids and 5′-nucleotides in cooked snail broth, accompanied by standardized sensory testing, would also allow the locally reported savory taste to be evaluated directly.

## 5. Conclusions

Open-air and greenhouse paddy systems were associated with different nutritional profiles in *C. hehuensis*, particularly for quantified fatty acids, vitamins, and selected elements. After Holm correction, 14 of the 19 quantified fatty acids differed, and ΣQFA was higher in open-air snails (1095.76 ± 7.02 vs. 817.63 ± 17.45 mg/kg WW; adjusted *p* = 0.004). Open-air snails had higher vitamin C, vitamin B3, and Fe and lower AI, TI, and n-3/n-6, whereas greenhouse snails had higher ash, vitamin A, Co, Al, and Pb. Moisture, crude protein, crude fat, and measured amino acids did not differ significantly, and acetophenone was the only significant assignment among 21 screened volatiles (adjusted *p* = 0.006). Together, these findings provide a baseline for nutritional characterization and highlight the need to monitor Al and Pb. Because samples came from one site and one harvest time, with three independent units per system and no paired dietary or environmental measurements, the associations should not be considered causal or generalized without further validation.

## Figures and Tables

**Table 1:** Amino acid composition and related indices (mg/100 g wet weight, except percentage ratios) of Hehu field snail muscle from open-air and greenhouse paddy culture systems.

Amino Acid or Index	Open-Air Paddy	Greenhouse Paddy	Adjusted *p*
Leu	76.2 ± 2.0	74.8 ± 0.9	1.000
Ile	44.1 ± 2.3	44.5 ± 0.1	1.000
Gly	195.1 ± 15.5	222.4 ± 51.4	1.000
Ala	115.7 ± 4.5	125.9 ± 2.1	0.900
Ser	169.2 ± 9.8	177.0 ± 7.8	1.000
Pro	51.3 ± 2.0	50.9 ± 0.8	1.000
Val	51.7 ± 2.0	50.9 ± 1.0	1.000
Thr	71.7 ± 3.5	80.9 ± 4.0	0.900
Asp	151.9 ± 6.1	146.6 ± 5.6	1.000
Lys	66.2 ± 2.4	65.0 ± 0.4	1.000
Glu	251.0 ± 10.9	247.2 ± 3.0	1.000
Met	18.2 ± 1.3	16.9 ± 0.4	1.000
His	15.9 ± 0.6	15.5 ± 0.5	1.000
Phe	40.1 ± 1.9	39.1 ± 0.2	1.000
Arg	46.2 ± 1.3	44.9 ± 0.8	1.000
Tyr	75.4 ± 3.4	76.5 ± 0.4	1.000
Cys	0.67 ± 0.08	0.69 ± 0.04	1.000
TAA	1440.5 ± 43.4	1479.8 ± 68.8	1.000
EAA	384.0 ± 15.5	387.8 ± 2.8	1.000
EAA/TAA (%)	26.65 ± 0.32	26.24 ± 1.06	1.000
DAA	713.7 ± 14.8	742.0 ± 59.1	1.000
DAA/TAA (%)	49.56 ± 0.79	50.09 ± 1.71	1.000

Values are means ± SD (n = 3 independent biological replicates per system), expressed as mg/100 g wet weight, except EAA/TAA and DAA/TAA, which are expressed as percentages. TAA, sum of quantified amino acids; EAA, Leu + Ile + Val + Thr + Lys + Met + Phe + His; DAA, Asp + Glu + Gly + Ala (acid-hydrolysate values). Trp was not measured (NM); Asn and Gln were not quantified separately (ND) under acid hydrolysis. Met and Cys may be underestimated. Holm-adjusted *p* values (22 two-sided Welch tests) are capped at 1.000.

**Table 2:** Concentrations of quantified fatty acids and calculated target-analyte sums in Hehu field snail muscle across culture systems (mg/kg wet weight). The n-3/n-6 ratio, AI, and TI are dimensionless; Σn-3 and Σn-6 are expressed in mg/kg wet weight.

Fatty Acid or Index	Open-Air Paddy	Greenhouse Paddy	Adjusted *p*
C4:0	70.39 ± 0.90	94.63 ± 7.48	0.144
C10:0	9.25 ± 0.59	7.96 ± 0.54	0.193
C11:0	16.13 ± 0.48	3.99 ± 0.11	0.004
C14:0	18.06 ± 0.44	17.94 ± 0.36	0.727
C15:0	21.29 ± 0.75	15.14 ± 0.26	0.020
C16:0	181.97 ± 1.71	140.52 ± 2.78	0.002
C16:1(cis-9)	16.11 ± 0.24	14.26 ± 0.24	0.007
C17:0	26.41 ± 0.92	19.52 ± 0.77	0.007
C18:0	95.84 ± 0.58	68.82 ± 1.75	0.006
C18:1(trans-9)	10.85 ± 0.16	13.60 ± 0.38	0.020
C18:1(cis-9)	87.07 ± 0.68	59.23 ± 1.40	0.002
C18:2(cis-9,12)	109.25 ± 1.55	69.34 ± 2.66	0.002
C18:3(cis-9,12,15)	23.69 ± 0.51	17.21 ± 0.71	0.005
C20:1(cis-11)	20.16 ± 0.77	13.32 ± 0.23	0.020
C21:0	16.33 ± 0.41	14.90 ± 0.82	0.227
C20:2(cis-11,14)	71.70 ± 1.05	41.51 ± 1.05	<0.001
C20:4(cis-5,8,11,14)	255.92 ± 3.31	172.07 ± 3.49	<0.001
C22:1(cis-13)	20.34 ± 0.86	11.37 ± 0.90	0.004
C20:5(cis-5,8,11,14,17) (EPA)	25.02 ± 0.40	22.31 ± 1.82	0.235
ΣQFA	1095.76 ± 7.02	817.63 ± 17.45	0.004
ΣQSFA	455.66 ± 2.41	383.41 ± 8.63	0.020
ΣQMUFA	154.53 ± 1.83	111.78 ± 1.95	<0.001
ΣQPUFA	485.57 ± 3.12	322.43 ± 7.50	0.002
ΣQUFA	640.10 ± 4.93	434.21 ± 9.26	0.001
QUFA/QSFA	1.40 ± 0.01	1.13 ± 0.01	<0.001
Σn-3 PUFA	48.70 ± 0.28	39.52 ± 2.30	0.038
Σn-6 PUFA	436.87 ± 3.41	282.92 ± 6.43	<0.001
n-3/n-6	0.111 ± 0.002	0.140 ± 0.008	0.038
AI	0.397 ± 0.004	0.489 ± 0.004	<0.001
TI	0.670 ± 0.006	0.719 ± 0.009	0.008

Values are means ± SD (n = 3 independent biological replicates per system). QUFA/QSFA is dimensionless. ΣQFA, sum of quantified fatty acids; ΣQSFA, saturated; ΣQMUFA, monounsaturated; ΣQPUFA, polyunsaturated; ΣQUFA, monounsaturated plus polyunsaturated. Sums and ratios were calculated per replicate from the 19 quantified targets. *p* values were obtained using two-sided Welch t tests, followed by separate Holm corrections for 25 fatty-acid composition comparisons (19 individual fatty acids and six summary measures) and five exploratory nutritional measures (Σn-3, Σn-6, n-3/n-6, AI, and TI). These five measures were derived from the quantified fatty acids only; n-3/n-6, AI, and TI are dimensionless. Accordingly, none of the reported fatty-acid sums should be interpreted as total fatty-acid content.

**Table 3:** Mineral element contents in the muscle of Hehu field snail across culture systems (dry-weight basis).

Element	Unit	Open-Air Paddy	Greenhouse Paddy	Adjusted *p*
**Macrominerals**				
Ca	g/kg	33.46 ± 0.60	31.20 ± 0.77	0.183
K	g/kg	4.54 ± 0.14	4.69 ± 0.11	1.000
Mg	g/kg	3.03 ± 0.15	3.66 ± 0.18	0.113
Na	g/kg	2.71 ± 0.13	2.57 ± 0.06	1.000
S	g/kg	6.12 ± 0.22	5.93 ± 0.16	1.000
**Trace and other elements**				
Fe	g/kg	2.87 ± 0.08	2.20 ± 0.12	0.030
Zn	mg/kg	230.00 ± 12.02	241.00 ± 11.20	1.000
Mn	mg/kg	63.53 ± 2.47	76.29 ± 3.05	0.067
Cu	mg/kg	68.94 ± 3.25	66.56 ± 3.05	1.000
Se	mg/kg	0.719 ± 0.033	0.768 ± 0.020	0.970
Co	mg/kg	0.623 ± 0.002	0.908 ± 0.026	0.033
Mo	mg/kg	0.384 ± 0.013	0.373 ± 0.030	1.000
B	mg/kg	117.63 ± 8.75	135.37 ± 14.12	1.000
Ge	mg/kg	0.817 ± 0.037	0.794 ± 0.021	1.000
Al	mg/kg	5560 ± 130	7240 ± 140	0.002
Pb	mg/kg	1.57 ± 0.06	2.32 ± 0.09	0.011

Values are means ± SD (n = 3 independent biological replicates per system). All contents are expressed on a dry-weight basis; units are specified for each element. *p* values were adjusted using the Holm method across 16 comparisons following two-sided Welch t tests.

**Table 4:** Comparison of 21 retained tentative volatile assignments in Hehu field snail muscle from open-air and greenhouse paddies.

Compound (Tentative)	Open-Air Paddy	Greenhouse Paddy	Adjusted *p*
Ethanol	271.571 ± 9.760	267.482 ± 2.039	1.000
Benzaldehyde	8.503 ± 0.258	4.404 ± 0.600	0.052
Octanal	2.238 ± 0.434	1.803 ± 0.083	1.000
Acetophenone	0.667 ± 0.023	0.403 ± 0.028	0.006
2-Nonanone	1.090 ± 0.055	0.748 ± 0.104	0.273
Nonanal	2.821 ± 0.033	2.239 ± 0.439	1.000
1H-Indene, 1-methylene-	0.510 ± 0.039	0.632 ± 0.128	1.000
2-Decanone	1.599 ± 0.041	1.190 ± 0.143	0.525
Dodecane	0.659 ± 0.095	0.573 ± 0.100	1.000
Decanal	1.293 ± 0.155	1.225 ± 0.109	1.000
2-Undecanone	1.180 ± 0.088	0.953 ± 0.235	1.000
Undecanal	1.799 ± 0.157	1.338 ± 0.239	0.929
Dodecanal	1.476 ± 0.045	1.082 ± 0.201	1.000
Pentadecane	0.764 ± 0.152	1.021 ± 0.212	1.000
Tridecanal	1.729 ± 0.169	1.473 ± 0.134	1.000
Dodecanoic acid	1.351 ± 0.068	1.263 ± 0.105	1.000
2-Tetradecanone	1.315 ± 0.140	0.932 ± 0.135	0.491
Hexadecane	1.709 ± 0.169	1.394 ± 0.314	1.000
2-Pentadecanone, 6,10,14-trimethyl-	2.410 ± 0.396	2.131 ± 0.646	1.000
Hexadecanoic acid, ethyl ester	1.691 ± 0.059	1.534 ± 0.457	1.000
2-Butenedioic acid (E)-, bis(2-ethylhexyl) ester	4.647 ± 0.848	4.740 ± 1.389	1.000

Values are raw integrated peak areas (×10^6^ counts·min; mean ± SD; n = 3 per system), not concentrations. Assignments are tentative and semi-quantitative (no internal standard/calibration). *p* values: Holm-adjusted Welch tests (21 comparisons). Full data: Table S2.

## Data Availability

The data presented in this study are available on request from the corresponding author.

## Supplementary Materials

The following supporting information can be downloaded at: https://www.mdpi.com/article/10.3390/foods15203743/s1, Table S1: Culture conditions and management practices of the open-air and greenhouse paddy systems during the production period before sampling; Table S2: Objective screening and statistical analysis of tentative HS-SPME-GC-MS assignments. Figure S1: Original GC-MS total ion chromatograms of the six paddy-culture samples.
