# Supplementary material for: Nutritional Composition of the Hehu Field Snail Cipangopaludina hehuensis: A Comparison of Open-Air and Greenhouse Paddy Culture Systems
Source: Foods. 2026 Oct 21;15(20):3743. doi: 10.3390/foods15203743 (PMC13704281; doi:10.3390/foods15203743)
Supplement: Supplementary file 1 [file foods-15-03743-s001.zip › foods-4627881-supplementary.pdf]

Supplementary Materials

Nutritional Composition of the Hehu Field Snail *Cipangopaludina hehuensis*: A Comparison of Open-Air and Greenhouse Paddy Culture Systems

Jie Chen, Xinyi Qian, Yikai Feng and Deju Zhang

Table S1. Culture conditions and management practices of the open-air and greenhouse paddy systems during the production period before sampling.

Parameter	Open-air paddy	Greenhouse paddy
Culture period before sampling	May–August 2025; approximately 90 d	May–August 2025; approximately 90 d
Holding unit and water depth	3 paddy-field units; 666.7 m^2 per unit; water depth approximately 0.30 m	3 greenhouse paddy-field units; 666.7 m^2 per unit; water depth approximately 0.30 m
Water exchange	Natural inflow and drainage	Natural inflow and drainage inside greenhouse
Water temperature	18.5–26.5 °C	21.5–29.0 °C
pH	6.8–7.6	7.1–8.1
Dissolved oxygen	5.5–8.0 mg/L	4.8–7.2 mg/L
Nutrient concentrations	NH_4^+-N 0.03–0.15 mg/L; NO_2^--N <0.05 mg/L; NO_3^--N 0.20–1.00 mg/L; TP 0.04–0.15 mg/L	NH_4^+-N 0.05–0.25 mg/L; NO_2^--N <0.05 mg/L; NO_3^--N 0.20–0.90 mg/L; TP 0.04–0.18 mg/L
Transparency and natural-food proxy	Transparency 25–45 cm; chlorophyll-a 20–45 µg/L; abundant periphyton, phytoplankton, detritus, and benthic microorganisms	Transparency 20–40 cm; chlorophyll-a 15–35 µg/L; moderate periphyton, phytoplankton, detritus, and benthic microorganisms
Stocking density	Approximately 5–6 seed snails/m^2 in paddy fields	Approximately 5–6 seed snails/m^2 in paddy fields
Feed input	Rice-bran-based mixed feed, approximately 1–2% of estimated snail biomass, supplied every 2–3 d when natural food was insufficient	Rice-bran-based mixed feed, approximately 1.5–2.5% of estimated snail biomass, supplied every 2–3 d
Feed composition	Rice bran 50%; wheat bran 20%; soybean meal or bean cake 20%; corn meal 10%	Rice bran 50%; wheat bran 20%; soybean meal or bean cake 20%; corn meal 10%
Fertilization and liming	Before stocking: water depth 12–18 cm; lime 30–50 kg/667 m^2; composted livestock manure 150–250 kg/667 m^2 as basal fertilizer	Before stocking: water depth 12–18 cm; lime 30–50 kg/667 m^2; composted livestock manure 150–250 kg/667 m^2 as basal fertilizer
Rice cultivation management	Rice transplanted in late May to early June at approximately 30 × 40 cm spacing; no molluscicides or herbicides; weeds and pests managed manually or ecologically	Rice transplanted in late May to early June at approximately 30 × 40 cm spacing; no molluscicides or herbicides; greenhouse ventilation and water-level control were used

Note: Values are production-period ranges or management records; both systems were located at the same Shuoyuan production site.

Table S2. Objective screening and statistical analysis of tentative HS-SPME-GC-MS assignments.

Screening summary: 368 unique compound/CAS identities were detected across the six paddy samples; 46 occurred in all six samples and 21 were retained for statistical testing.

Screening criteria: detection in all six samples, a valid CAS number, reverse-library-match score ≥800 in every sample, and exclusion of common siloxane, phthalate, and phosphate-ester backgrounds.

Table S2A. Identification, library-match quality, and screening decisions for assignments present in all six samples.

Compound	CAS	Formula	Mean RT (min)	Min forward match	Min reverse match	Decision
Ethanol	64-17-5	C_2H_6O	1.890	855	861	Included
Heptanal	111-71-7	$C_7H_{14}O$	7.264	711	792	Excluded: reverse match <800
Benzaldehyde	100-52-7	C_7H_6O	8.745	865	899	Included
1-Octen-3-ol	3391-86-4	$C_8H_{16}O$	9.224	638	788	Excluded: reverse match <800
Cyclotetrasiloxane, octamethyl-	556-67-2	$C_8H_{24}O_4Si_4$	9.667	813	873	Excluded: siloxane background
Octanal	124-13-0	$C_8H_{16}O$	9.810	671	809	Included
Acetophenone	98-86-2	C_8H_8O	11.465	790	876	Included
2-Nonanone	821-55-6	$C_9H_{18}O$	12.076	737	868	Included
Nonanal	124-19-6	$C_9H_{18}O$	12.388	866	871	Included
Cyclopentasiloxane, decamethyl-	541-02-6	$C_{10}H_{30}O_5Si_5$	13.609	846	852	Excluded: siloxane background
1H-Indene, 1-methylene-	2471-84-3	$C_{10}H_8$	14.458	748	915	Included
2-Decanone	693-54-9	$C_{10}H_{20}O$	14.571	825	858	Included
Dodecane	112-40-3	$C_{12}H_{26}$	14.761	824	881	Included
Decanal	112-31-2	$C_{10}H_{20}O$	14.893	841	851	Included
Nonanoic acid	112-05-0	$C_9H_{18}O_2$	16.425	751	797	Excluded: reverse match <800
Pentasiloxane, 1,1,3,3,5,5,7,7,9,9-decamethyl-	995-83-5	$C_{10}H_{32}O_4Si_5$	16.788	651	694	Excluded: siloxane background
2-Undecanone	112-12-9	$C_{11}H_{22}O$	16.955	855	865	Included
Undecanal	112-44-7	$C_{11}H_{22}O$	17.268	849	937	Included
Cyclohexasiloxane, dodecamethyl-	540-97-6	$C_{12}H_{36}O_6Si_6$	17.671	840	847	Excluded: siloxane background
2-Undecanone, 6,10-dimethyl-	1604-34-8	$C_{13}H_{26}O$	19.198	452	607	Excluded: reverse match <800
2-Dodecanone	6175-49-1	$C_{12}H_{24}O$	19.210	704	788	Excluded: reverse match <800
Dodecanal	112-54-9	$C_{12}H_{24}O$	19.431	650	829	Included
5,9-Undecadien-2-one, 6,10-dimethyl-, (E)-	3796-70-1	$C_{13}H_{22}O$	20.452	708	766	Excluded: reverse match <800
Cycloheptasiloxane, tetradecamethyl-	107-50-6	$C_{14}H_{42}O_7Si_7$	21.361	759	777	Excluded: siloxane background
Pentadecane	629-62-9	$C_{15}H_{32}$	21.423	858	875	Included
Tridecanal	10486-19-8	$C_{13}H_{26}O$	21.647	853	876	Included
2(4H)-Benzofuranone, 5,6,7,7a-tetrahydro-4,4,7a-trimethyl-, (R)-	17092-92-1	$C_{11}H_{16}O_2$	22.207	662	772	Excluded: reverse match <800
Phosphoric acid, diethyl octyl ester		$C_{12}H_{27}O_4P$	22.353	710	744	Excluded: phosphate-ester background
Dodecanoic acid	143-07-7	$C_{12}H_{24}O_2$	22.686	785	834	Included
2-Tetradecanone	2345-27-9	$C_{14}H_{28}O$	23.365	799	851	Included
Hexadecane	544-76-3	$C_{16}H_{34}$	23.408	820	863	Included
Cyclooctasiloxane, hexadecamethyl-	556-68-3	$C_{16}H_{48}O_8Si_8$	24.668	815	837	Excluded: siloxane background
EMDP	102177-18-4	$C_{19}H_{21}N$	25.352	618	702	Excluded: reverse match <800
Tetradecanoic acid	544-63-8	$C_{14}H_{28}O_2$	26.403	707	761	Excluded: reverse match <800
Tetradecanoic acid, ethyl ester	124-06-1	$C_{16}H_{32}O_2$	26.976	702	773	Excluded: reverse match <800
2-Pentadecanone, 6,10,14-trimethyl-	502-69-2	$C_{18}H_{36}O$	27.865	799	859	Included
Phthalic acid, butyl tetradecyl ester		$C_{26}H_{42}O_4$	28.306	756	822	Excluded: phthalate background
Hexadecanoic acid, ethyl ester	628-97-7	$C_{18}H_{36}O_2$	30.320	806	830	Included
Octadecane, 3-ethyl-5-(2-ethylbutyl)-	55282-12-7	$C_{26}H_{54}$	30.361	464	487	Excluded: reverse match <800
Octasiloxane, 1,1,3,3,5,5,7,7,9,9,11,11,13,13,15,15-hexadecamethyl-	19095-24-0	$C_{16}H_{50}O_7Si_8$	30.440	523	667	Excluded: siloxane background
Cyclohexane, 1,1'-dodecylidenebis[4-methyl-	55334-09-3	$C_{26}H_{50}$	30.908	485	514	Excluded: reverse match <800

Compound	CAS	Formula	Mean RT (min)	Min forward match	Min reverse match	Decision
17-Pentatriacontene	6971-40-0	$C_{35}H_{70}$	30.933	461	479	Excluded: reverse match <800
Eicosane	112-95-8	$C_{20}H_{42}$	32.008	668	773	Excluded: reverse match <800
Octatriacontyl pentafluoropropionate		$C_{41}H_{77}F_5O_2$	32.280	607	644	Excluded: no valid CAS
Tetrapentacontane, 1,54-dibromo-		$C_{54}H_{108}Br_2$	32.621	537	559	Excluded: no valid CAS
2-Butenedioic acid (E)-, bis(2-ethylhexyl) ester	141-02-6	$C_{20}H_{36}O_4$	33.923	801	829	Included

Table S2A (continued). Six-sample raw integrated peak areas used in prevalence screening (×10^6 counts·min).

Compound	CAS	C2-1 area (×10^6)	C2-2 area (×10^6)	C2-3 area (×10^6)	C3-1 area (×10^6)	C3-2 area (×10^6)	C3-3 area (×10^6)
Ethanol	64-17-5	278.811	275.429	260.472	268.574	268.743	265.130
Heptanal	111-71-7	1.349	1.260	0.803	0.826	1.387	0.673
Benzaldehyde	100-52-7	8.240	8.515	8.755	4.868	3.726	4.617
1-Octen-3-ol	3391-86-4	1.869	0.990	1.011	0.574	0.521	0.588
Cyclotetrasiloxane, octamethyl-	556-67-2	1.014	1.558	1.064	0.950	0.830	1.028
Octanal	124-13-0	1.972	2.004	2.739	1.712	1.874	1.824
Acetophenone	98-86-2	0.655	0.653	0.693	0.435	0.391	0.382
2-Nonanone	821-55-6	1.150	1.040	1.082	0.857	0.735	0.651
Nonanal	124-19-6	2.844	2.782	2.836	2.683	2.228	1.805
Cyclopentasiloxane, decamethyl-	541-02-6	9.282	7.278	10.247	6.604	8.166	6.654
1H-Indene, 1-methylene-	2471-84-3	0.494	0.481	0.554	0.728	0.681	0.486
2-Decanone	693-54-9	1.637	1.557	1.604	1.317	1.219	1.034
Dodecane	112-40-3	0.572	0.644	0.761	0.634	0.626	0.457
Decanal	112-31-2	1.204	1.202	1.471	1.316	1.255	1.103
Nonanoic acid	112-05-0	0.756	0.744	1.211	1.273	1.080	1.006
Pentasiloxane, 1,1,3,3,5,5,7,7,9,9-decamethyl-	995-83-5	0.550	0.558	0.270	0.280	0.254	0.263
2-Undecanone	112-12-9	1.188	1.088	1.263	1.219	0.868	0.772
Undecanal	112-44-7	1.763	1.664	1.971	1.614	1.192	1.208
Cyclohexasiloxane, dodecamethyl-	540-97-6	25.301	20.238	30.940	20.259	25.237	22.044
2-Undecanone, 6,10-dimethyl-	1604-34-8	1.392	1.768	2.285	1.476	1.528	0.953
2-Dodecanone	6175-49-1	0.907	0.673	0.801	1.137	1.064	0.909
Dodecanal	112-54-9	1.468	1.525	1.436	1.242	1.148	0.856
5,9-Undecadien-2-one, 6,10-dimethyl-, (E)-	3796-70-1	0.558	0.502	0.530	0.415	0.370	0.310
Cycloheptasiloxane, tetradecamethyl-	107-50-6	15.276	12.852	17.349	12.128	13.182	12.400
Pentadecane	629-62-9	0.640	0.718	0.933	0.809	1.234	1.019
Tridecanal	10486-19-8	1.607	1.659	1.921	1.567	1.533	1.320
2(4H)-Benzofuranone, 5,6,7,7a-tetrahydro-4,4,7a-trimethyl-, (R)-	17092-92-1	0.357	0.344	0.387	0.266	0.297	0.228
Phosphoric acid, diethyl octyl ester		1.606	1.898	1.866	2.219	2.098	1.786
Dodecanoic acid	143-07-7	1.300	1.325	1.428	1.164	1.251	1.373
2-Tetradecanone	2345-27-9	1.185	1.296	1.464	1.061	0.944	0.791
Hexadecane	544-76-3	1.638	1.587	1.902	1.741	1.309	1.131
Cyclooctasiloxane, hexadecamethyl-	556-68-3	3.964	3.930	4.723	3.305	3.747	3.396
EMDP	102177-18-4	0.211	1.044	1.443	0.577	0.243	0.747
Tetradecanoic acid	544-63-8	0.712	1.571	2.025	2.076	1.011	1.658
Tetradecanoic acid, ethyl ester	124-06-1	0.730	0.697	0.779	0.700	0.737	0.655
2-Pentadecanone, 6,10,14-trimethyl-	502-69-2	2.030	2.381	2.819	2.805	2.073	1.517
Phthalic acid, butyl tetradecyl ester		1.317	1.425	0.922	1.804	1.600	1.252
Hexadecanoic acid, ethyl ester	628-97-7	1.759	1.646	1.670	1.593	1.959	1.051
Octadecane, 3-ethyl-5-(2-ethylbutyl)-	55282-12-7	5.525	5.444	3.775	2.612	3.021	4.000
Octasiloxane, 1,1,3,3,5,5,7,7,9,9,11,11,13,13,15,15-hexadecamethyl-	19095-24-0	1.503	3.522	1.702	3.643	3.172	1.307
Cyclohexane, 1,1'-dodecylidenebis[4-methyl-	55334-09-3	0.391	0.901	0.228	0.991	0.179	0.897

Compound	CAS	C2-1 area (×10⁶)	C2-2 area (×10⁶)	C2-3 area (×10⁶)	C3-1 area (×10⁶)	C3-2 area (×10⁶)	C3-3 area (×10⁶)
17-Pentatriacontene	6971-40-0	5.908	9.293	4.355	7.095	7.430	7.581
Eicosane	112-95-8	39.773	20.696	48.850	48.622	22.780	23.308
Octatriacontyl pentafluoropropionate		4.095	1.946	1.149	4.458	1.606	3.521
Tetrapentacontane, 1,54-dibromo-		14.767	18.153	20.710	15.152	24.025	6.185
2-Butenedioic acid (E)-, bis(2-ethylhexyl) ester	141-02-6	4.284	4.041	5.616	5.962	5.030	3.229

C2-1 to C2-3 are the open-air paddy replicates; C3-1 to C3-3 are the greenhouse paddy replicates. Repeated peaks with the same CAS number were summed within sample.

Table S2B. Raw peak areas of the 21 objectively screened tentative assignments ($\times 10^6$ counts·min).

Compound	CAS	Mean RT (min)	C2-1 (×10⁶)	C2-2 (×10⁶)	C2-3 (×10⁶)	C3-1 (×10⁶)	C3-2 (×10⁶)	C3-3 (×10⁶)
Ethanol	64-17-5	1.890	278.811	275.429	260.472	268.574	268.743	265.130
Benzaldehyde	100-52-7	8.745	8.240	8.515	8.755	4.868	3.726	4.617
Octanal	124-13-0	9.810	1.972	2.004	2.739	1.712	1.874	1.824
Acetophenone	98-86-2	11.465	0.655	0.653	0.693	0.435	0.391	0.382
2-Nonanone	821-55-6	12.076	1.150	1.040	1.082	0.857	0.735	0.651
Nonanal	124-19-6	12.388	2.844	2.782	2.836	2.683	2.228	1.805
1H-Indene, 1-methylene-	2471-84-3	14.458	0.494	0.481	0.554	0.728	0.681	0.486
2-Decanone	693-54-9	14.571	1.637	1.557	1.604	1.317	1.219	1.034
Dodecane	112-40-3	14.761	0.572	0.644	0.761	0.634	0.626	0.457
Decanal	112-31-2	14.893	1.204	1.202	1.471	1.316	1.255	1.103
2-Undecanone	112-12-9	16.955	1.188	1.088	1.263	1.219	0.868	0.772
Undecanal	112-44-7	17.268	1.763	1.664	1.971	1.614	1.192	1.208
Dodecanal	112-54-9	19.431	1.468	1.525	1.436	1.242	1.148	0.856
Pentadecane	629-62-9	21.423	0.640	0.718	0.933	0.809	1.234	1.019
Tridecanal	10486-19-8	21.647	1.607	1.659	1.921	1.567	1.533	1.320
Dodecanoic acid	143-07-7	22.686	1.300	1.325	1.428	1.164	1.251	1.373
2-Tetradecanone	2345-27-9	23.365	1.185	1.296	1.464	1.061	0.944	0.791
Hexadecane	544-76-3	23.408	1.638	1.587	1.902	1.741	1.309	1.131
2-Pentadecanone, 6,10,14-trimethyl-	502-69-2	27.865	2.030	2.381	2.819	2.805	2.073	1.517
Hexadecanoic acid, ethyl ester	628-97-7	30.320	1.759	1.646	1.670	1.593	1.959	1.051
2-Butenedioic acid (E)-, bis(2-ethylhexyl) ester	141-02-6	33.923	4.284	4.041	5.616	5.962	5.030	3.229

Table S2B (continued). Group summaries and exploratory statistical tests for the 21 screened assignments.

Compound	Open-air mean	Open-air SD	Greenhouse mean	Greenhouse SD	Open/greenhouse ratio	Welch P	Holm-adjusted P
Ethanol	271.571	9.760	267.482	2.039	1.015	0.5460	1
Benzaldehyde	8.503	0.258	4.404	0.600	1.931	0.0026	0.0516
Octanal	2.238	0.434	1.803	0.083	1.241	0.2214	1
Acetophenone	0.667	0.023	0.403	0.028	1.657	0.0003	0.0059
2-Nonanone	1.090	0.055	0.748	0.104	1.458	0.0144	0.2731
Nonanal	2.821	0.033	2.239	0.439	1.260	0.1479	1
1H-Indene, 1-methylene-	0.510	0.039	0.632	0.128	0.807	0.2362	1
2-Decanone	1.599	0.041	1.190	0.143	1.344	0.0309	0.5249
Dodecane	0.659	0.095	0.573	0.100	1.151	0.3391	1
Decanal	1.293	0.155	1.225	0.109	1.055	0.5722	1
2-Undecanone	1.180	0.088	0.953	0.235	1.238	0.2318	1
Undecanal	1.799	0.157	1.338	0.239	1.345	0.0580	0.9288
Dodecanal	1.476	0.045	1.082	0.201	1.364	0.0703	1
Pentadecane	0.764	0.152	1.021	0.212	0.748	0.1702	1

Compound	Open-air mean	Open-air SD	Greenhouse mean	Greenhouse SD	Open/greenhouse ratio	Welch P	Holm-adjusted P
Tridecanal	1.729	0.169	1.473	0.134	1.174	0.1123	1
Dodecanoic acid	1.351	0.068	1.263	0.105	1.070	0.2991	1
2-Tetradecanone	1.315	0.140	0.932	0.135	1.411	0.0273	0.4912
Hexadecane	1.709	0.169	1.394	0.314	1.226	0.2211	1
2-Pentadecanone, 6,10,14-trimethyl-	2.410	0.396	2.131	0.646	1.131	0.5657	1
Hexadecanoic acid, ethyl ester	1.691	0.059	1.534	0.457	1.102	0.6134	1
2-Butenedioic acid (E)-, bis(2-ethylhexyl) ester	4.647	0.848	4.740	1.389	0.980	0.9268	1

P values are from two-sided Welch t tests on raw integrated peak areas; Holm adjustment was applied across the 21 screened assignments.

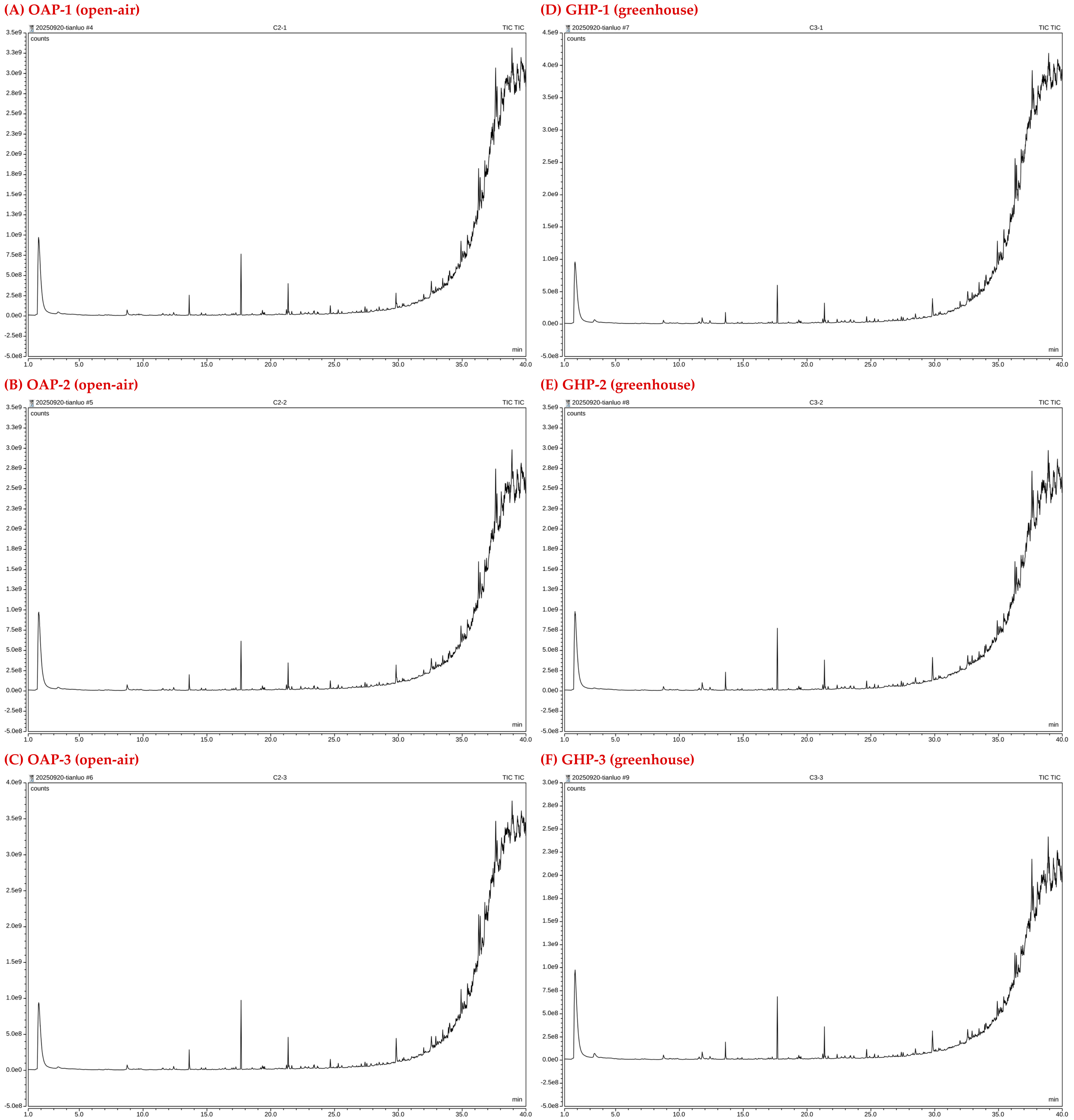

Figure S1. HS-SPME-GC-MS total ion chromatograms (TICs) of edible muscle samples from open-air paddy (A – C: OAP-1, OAP-2, and OAP-3) and greenhouse paddy (D – F: GHP-1, GHP-2, and GHP-3) culture systems. The chromatographic signals represent instrument responses rather than calibrated compound concentrations.
